# Stimuli Followed by Avian Malaria Vectors in Host-Seeking Behaviour

**DOI:** 10.3390/biology11050726

**Published:** 2022-05-09

**Authors:** Alfonso Marzal, Sergio Magallanes, Luz Garcia-Longoria

**Affiliations:** 1 Department of Anatomy, Cellular Biology and Zoology, University of Extremadura, Avenida de Elvas s/n, 06006 Badajoz, Spain; luzlongoria@unex.es; 2 Grupo de Investigación y Sostenibilidad Ambiental, Universidad Nacional Federico Villarreal, Lima 15007, Peru; 3 Department of Wetland Ecology, Biological Station (EBD-CSIC), Avda, Américo Vespucio 26, 41092 Sevilla, Spain; sergioma@unex.es

**Keywords:** haemosporidian, mosquitoes, parasite manipulation hypothesis, preen oil, vector attractants

## Abstract

**Simple Summary:**

Vector-borne diseases (VBDs) (e.g., malaria, yellow fever, dengue fever) account for 17% of the estimated global burden of all infectious diseases. They are transmitted to humans and other animals by blood-feeding arthropods. In their pursuit of blood meal, insect vectors use different cues to detect their hosts. The knowledge of these stimuli followed by vectors in this host-seeking behaviour is essential to design strategies to prevent VBD infections. Since its discovery in the late 19th century, avian malaria investigations have allowed significant advances to understand the dynamics and mechanisms of VBD transmission to many organisms, including humans. Here, we review published contributions on the different physical and chemical cues used by mosquitoes and other bird haemosporidian vectors to locate their hosts. This information would be highly valuable for vector surveillance and public health policies.

**Abstract:**

Vector-borne infectious diseases (e.g., malaria, dengue fever, and yellow fever) result from a parasite transmitted to humans and other animals by blood-feeding arthropods. They are major contributors to the global disease burden, as they account for nearly a fifth of all infectious diseases worldwide. The interaction between vectors and their hosts plays a key role driving vector-borne disease transmission. Therefore, identifying factors governing host selection by blood-feeding insects is essential to understand the transmission dynamics of vector-borne diseases. Here, we review published information on the physical and chemical stimuli (acoustic, visual, olfactory, moisture and thermal cues) used by mosquitoes and other haemosporidian vectors to detect their vertebrate hosts. We mainly focus on studies on avian malaria and related haemosporidian parasites since this animal model has historically provided important advances in our understanding on ecological and evolutionary process ruling vector-borne disease dynamics and transmission. We also present relevant studies analysing the capacity of feather and skin symbiotic bacteria in the production of volatile compounds with vector attractant properties. Furthermore, we review the role of uropygial secretions and symbiotic bacteria in bird–insect vector interactions. In addition, we present investigations examining the alterations induced by haemosporidian parasites on their arthropod vector and vertebrate host to enhance parasite transmission. Finally, we propose future lines of research for designing successful vector control strategies and for infectious disease management.

## 1. Avian Haemosporidians and Their Vectors

Vector-borne diseases (e.g., malaria, yellow fever, dengue fever) are major contributors to the global disease burden. Malaria is probably the most deathly and prevalent parasitic disease in the history of mankind. Indeed, it is estimated that about 150–300 million people have died from the effects of malaria during the past 100 years [1]. In 2020, there were an estimated 241 million cases of malaria worldwide, and 40% of the world’s population still lives in areas where malaria is transmitted [2].

However, the systematicity and diversity of malaria parasites is much larger and not restricted to human parasites. These protozoan intracellular pathogens belong to order Haemosporidia, with numerous species from 15 genera infecting reptiles, birds, and mammals all around the world [3]. Avian haemosporidians are the largest group among all the haemosporidians infecting vertebrates by number of described species [4,5]. So far, more than 4600 parasite lineages from the genera *Plasmodium*, *Haemoproteus*, *Leucocytozoon*, and *Fallisia* have been described in more than 1900 avian species (MALAVI database version 2.5.2, December 2021 [6]). Moreover, new lineages are reported every year revealing the remaining unexplored genetic diversity of these parasites, mainly in the tropics [7,8,9,10]. These blood parasites may provoke detrimental effects on their avian host by reducing their survival [11,12,13], minimizing their reproductive success [14,15] and provoking tissue damage [16], hence reducing bird populations and eventually being responsible for population extinctions following the introduction of exotic haemosporidian parasites beyond their natural range [17]. They are globally distributed, infecting individuals representing most bird clades in all the continents except Antarctica [18], thus constituting an excellent model for the study of vector-host–parasite interactions [4].

The term “malaria parasites” has been a debated issue among parasitologists, ecologists, and evolutionary researchers [19,20]. The controversy lies from the incomplete knowledge of the phylogenetic relationships and pathogenicity of non-human malaria parasites [21]. Although some similarities can be observed in the life cycles of *Plasmodium*, *Haemoproteus*, and *Leucocytozoon*, they still have some differences in vectors, life cycles, and epidemiology [22]. Therefore, traditional taxonomists and parasitologists only accept *Plasmodium* species as being the true malaria parasites [4]. However, based on molecular genetic studies describing the phylogeny of the group, other authors also include other genera (i.e., *Haemoproteus*, *Leucocytozoon*) among the term “malaria parasites” [19]. Haemosporidians are obligate heteroxenous parasites, with some parts of their life cycle developing within their blood-feeding arthropod vectors (sexual reproduction), whereas some stages occur within their vertebrate hosts (asexual reproduction). After the inoculation of haemosporidian sporozoites from an infective vector, the parasites may either complete their life cycle in a susceptible host or abort their development in a non-susceptible host unable to develop infective stages (gametocytes) to reach a new host [5].

The infection starts with the bite of a female dipteran insect transmitting infective stages (sporozoites) from its saliva into the blood stream of the avian host while taking a blood meal. Afterwards, the sporozoites initiate the development of exoerythrocytic meronts in the endothelial cells of many organs and tissues. Meronts undergo asexual divisions in these cells and form merozoites for a minimum of two generations before the parasite produce merozoites capable to infect erythrocytes. This part of the life cycle before the development of merozoites that are able of invading blood cells is called the prepatent period (10–14 days). This extraerythrocytic stage is essential to enhance the initial infectious source. The breakage of host endothelial cells releases merozoites into the blood stream, which may result in (i) additional infection of reticuloendothelial cells; or (ii) invasion of red blood cells giving rise to gametocytes (macrogametocytes and microgametocytes), which are infective to vectors. Gametocytes remain inside erythrocytes until ingestion by a dipteran insect in which the sexual process and sporogony take place. The inoculation of infective sporozoites will initiate new infections in vertebrate hosts [4,5,22].

The patent period of infection (interval during which parasites can be found in the blood stream) begins when parasites enter circulating erythrocytes, and encompasses different phases: (a) the acute stage, the initial phase when intensity of parasitaemia increases; (b) crisis, when parasitaemia reaches a maximum; and (c) the chronic phase, where the parasitaemia decreases and stabilizes at low levels. In haemosporidian infection, however, the chronic phase may be followed by a latent stage of infection, where parasites are absent in the blood stream but persist in internal organs. These tissue stages may initiate asexual replications leading to relapses and temporary increases of parasitaemia [4,5,22]. It has been shown that avian malaria *Plasmodium relictum* reacts to mosquito bites by increasing its overall parasitaemia in the blood during the chronic stage of the infection, which may result in enhanced probability of infection to mosquitoes and thus increased transmission rates [23].

To date, only species of blood-sucking dipteran insects (Diptera) have been described as vectors for haemosporidian parasites [24]. Culicidae mosquitoes from five genera (*Anopheles*, *Culex*, *Aedes*, *Culiseta*, *Coquillettidia*) are capable of transmitting avian *Plasmodium* parasites [24]. Other mosquito genera such as *Mansonia* and *Lutzia* have been found to carry *Plasmodium* lineages [25,26], but their competence in successfully transmitting malaria parasites still needs experimental confirmation (e.g., visual and molecular identification of sporozoites in salivary glands of these mosquitoes). Within the genus *Haemoproteus*, biting midges (mostly of the genus *Culicoides*, Ceratopogonidae) transmit parasites of the subgenus *Parahaemoproteus*, whereas parasites from subgenus *Haemoproteus* are vectored by louse flies (Hippoboscidae) [24]. For the genus *Leucocytozoon*, it is generally accepted that parasite species from subgenus *Leucocytozoon* are transmitted by black flies (Simuliidae), while ceratopogonid flies are responsible for the transmission of the only species of *Akiba* subgenus infecting birds (*A. caulleryi*) [27]. The only species of the genus *Fallisia* infecting birds is supposed to be transmitted by culicine mosquitoes [28], but this requires verification.

## 2. Cues Followed by Haemosporidian Vectors to Locate Their Hosts

**Figure 1:**
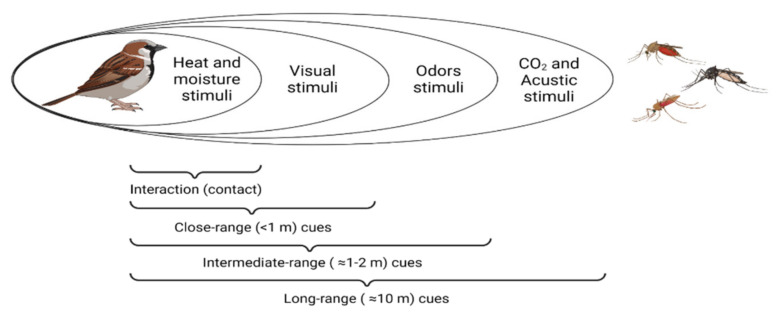
The sensory cues used by mosquitoes to detect their prey are distance-dependent. Mosquitoes follow a combination of cues to detect their potential hosts according to their proximity. Mosquitoes at larger distances can detect odours and CO_2_ exhaled from host’s breath, whereas vectors use body temperature and visual stimuli to locate their hosts at closer ranges. Adapted from [34,35].

Vector control is a crucial strategy for global malaria control in preventing infection and reducing disease transmission [29]. Although the contact between hosts and vectors may play a key role driving vector-borne disease transmission, vector density has been largely studied to analyse transmission risk, while host–vector contact dynamics, including host-seeking behaviour, have received less attention [30]. Historically, avian models have provided important insights to explain variations in disease risk, thus enhancing our knowledge on ecological and evolutionary processes ruling host–parasite interactions [31]. Identifying factors governing host selection by blood-feeding insects is essential to understand the transmission dynamics of vector-borne diseases [32]. Arthropod vectors may use a number of physical and chemical stimuli emitted by vertebrate hosts to detect their blood meal sources, including acoustic, visual, olfactory, moisture, and thermal cues (Figure 1) [33,34,35]. Next, we detail the different cues used by mosquitoes and other haemosporidian vectors to locate their hosts, with special emphasis on the role of uropygial gland secretion on the bird–malaria vector interaction (Table 1).

### 2.1. Visual Stimuli

Adult mosquitoes possess compound eyes that are sensitive to high-contrast visual features such as colours, varying light intensity, host pattern, and motion [57]. These visual signals are considered to be important stimuli in the activation, orientation, and landing of blood-feeding insects [34] and may act synergically with other stimuli. For example, tracking studies and behavioural observations have revealed that the African malaria vector (*Anopheles gambiae*) and the yellow fever mosquito (*Aedes aegypti*) activated host-seeking behaviour and perform upwind flights at long distance in response to detected olfactory signals (human-emanated CO_2_), but rely on visual cues at intermediate distances, and start landing when in contact with close-range host hints, such as body heat and humidity [58,59,60,61].

Several features have been suggested to act as visual stimuli for avian haemosporidian vectors. Despite many mosquito species performing host-seeking behaviour during crepuscular or nocturnal hours [62], there is some evidence indicating that they can detect visual cues even in darkness [63]. In these poor visibility conditions, light colours attract more mosquitoes than dark colours [64,65], hence suggesting that colour/intensity contrast against background plays a role in vector attraction. By analysing blood feeding patterns in *Culex pipiens* for North American bird species, Yan et al. [36] found that this mosquito vector is more attracted to birds with a greater colour contrast against the background. In addition, mosquito compound eyes are very sensitive to motion and may detect movements, thereby facilitating host location. In support for this idea, Tomás et al. [37] explored the factors that may affect the abundance of biting midges in the nest cavity of blue tit (*Cyanistes caeruleus*). They showed that the abundance of biting midges was positively associated with parental provisioning effort, which could make their nests easier to locate by these dipteran vectors because the increased motion activity of parents during nestling feeding period.

Some other studies have explored the relationship between vector feeding preference and body size/mass in birds, revealing that larger birds showed increased attraction of some blood-sucking dipterans, such as mosquitoes [66], biting midges [67], and blackflies [68]. In this line, Yan et al. [36] investigated the relationship between some phenotypic traits related to body size (mean body mass, tarsus length, and bill length) and blood-feeding patterns in *Cx. pipiens* and *Culex restuans* on a North American avian community. They showed that these vector species fed preferably on birds with longer tarsi, suggesting that mosquitoes may have greater attraction towards larger birds. This pattern of feeding preference of avian haemosporidian vectors for larger birds has also been proposed by Ganser et al. [33] to explain the higher *Leucocytoozon* prevalence in Guinean fowl and doves when analysing the association between body size and prevalence of three haemosporidian genera in 17 savannah bird species from Africa. Because larger-bodied birds produce more olfactory cues used by dipteran vector to locate blood meals [69], the positive relationship between body size/mass and increased attraction of blood-sucking dipterans does not exclude the alternative idea that avian haemosporidian vectors rely on host-derived chemicals such as ammonia, lactic acid, and carbon dioxide to locate blood meals [70]. These outcomes suggest that the integration of different sensory cues by vectors is necessary to make robust decisions in host-seeking behaviour.

### 2.2. Heat and Moisture Stimuli

Other physical cues, such as heat and humidity, could also be perceived by female mosquitoes and play a role in short-range attraction. During the pursuit of a blood meal, mosquitoes can identify the presence of the hosts because they are attracted to the heat produced by the metabolic activity of the host [34]. Therefore, vector thermoreceptors are used to locate and feed on warm-blooded hosts, as it has been shown in some species of mosquitoes such as *Ae. aegypti* and *An. gambiae* [71,72]. Since body heat becomes attractive in the proximity to the host (usually at distances shorter than 1 m) [59,60], this thermal sensory stimulus usually occurs during the landing phase of host-seeking, and with simultaneous activation of olfactory and/or visual receptors [73]. In support for this idea, it has been shown that vector traps also baited with a heat source significantly increased the number of captured mosquitoes [74,75]. For example, *Ae. aegypti* is only attracted to model human when heat source is in close vicinity [60]. Moreover, Hawkes et al. [74] quantified *Anopheles* responses to olfactory, visual, and thermal stimuli using a simple adhesive trap, showing that a trap combining odour and visual hints with a heat signature in the range equivalent to human body temperature significantly captured more mosquitoes than other traps without thermal stimuli. Moreover, Martínez de la Puente et al. [38] found that the abundance of biting midges increased with temperature inside the nest of pied flycatchers (*Ficedula hypoleuca*), which may suggest that nest temperature could be a cue used by insects to localize their hosts.

However, outcomes for experimental studies in birds are not so straightforward when testing the vector-biting rate of individuals with different body heats. For example, Cozzarolo et al. [39] performed host choice behaviour experiments by simultaneously presenting female *Cx. pipens* mosquitoes to a 14 day-old male and female great tit (*Parus major*). Surprisingly, nestling great tits with lower temperature were bitten more frequently by mosquitoes independently of their sex. Similarly, Yan et al. [40] explored the hypothesis that mosquitoes would prefer to feed on a host with higher metabolic rates, because they would produce more stimuli for host-seeking vectors. They manipulated the resting metabolic rate of house sparrows and then subsequently exposed them to *Cx. pipiens* mosquitoes to analyse the blood-feeding preference of vectors. Contrary to their expectations, they found that sparrows with lower resting metabolic rate suffered more mosquito bites than birds with higher resting metabolic range, thus suggesting that host metabolic rate does affect mosquito feeding preference. These apparent discrepancies in the outcomes from observational and experimental studies can be explained because mosquitoes are attracted to thermal stimuli emitted by their hosts, but other factors (e.g., host activity, anti-mosquito behaviour) may modulate biting rates.

Mosquitoes, simulids, and biting midges are also sensitive to variations in moisture and may perform avoidance or attraction reactions towards variations in relative humidity [76,77,78]. Therefore, humidity has also been suggested to act as a synergic cue in combination with other stimuli in short-range host seeking behaviour. However, there are mixed and inconclusive results among studies when analysing the attraction for vectors to humidity sources. For example, heat and moisture did not influence the relative attractiveness of the odour-bait in a dual-port olfactometer bioassay testing the behavioural responses of female *An. gambiae* mosquitoes towards attractants [79]. In contrast, other study in laboratory and semi-field conditions showed that the number of captured female *Anopheles coluzzii* and *An. gambiae* significantly increased when some short-range host cues (e.g., heat and humidity) were added to odour-baited traps [80]. Determining the impact of humidity on the flight activity of *Culicoides imicola*, Venter et al. [81] found no correlation between relative humidity and flight activity under laboratory conditions. They concluded that in field conditions relative humidity is correlated with temperature, although it seems to play a secondary role in flight initiation in biting midges. Moreover, Castaño-Vázquez et al. [41] did not find any evidence of change in the abundance of biting midges and black flies in the blue tit (*Cyanistes caeruleus*) nests with experimentally increased in temperature (3 °C on average) and a reduction in relative humidity (of about six units), thus suggesting that higher temperatures may not facilitate the detection of nests by haemosporidian vectors. However, since ectoparasite development is adapted to an optimum temperature-humidity range [82], the experimental increase in temperature and associated decrease in moisture may explain the lower abundance of ectoparasites in heated blue tits nests.

### 2.3. Acoustic Stimuli

The fast transmission of auditory signals is highly beneficial for information receiver to accurately find out the sound source (i.e., ectoparasites to locate their hosts). Moreover, these cues can be used simultaneously and in redundancy with other sensory stimuli (e.g., visual or olfactory stimuli) to reinforce the messages [83]. Insects possess a highly efficient auditory system, which may facilitate host seeking and location when environmental conditions constrain the use of other stimuli, such as limited efficiency of visual cues due to reduced light availability at night or the absence of wind minimizing the use of olfactory signals [84]. Therefore, it has been proposed that arthropod vectors may use auditory cues, probably in combination with other host hints, for host location [85]. In support of this hypothesis, some studies have shown that blood-feeding ectoparasites of amphibians such as biting midges and Culicidae mosquitoes are attracted to frog calls [86].

However, the capacity of ectoparasites to be attracted towards bird sounds remains quite unexplored (see a recent review by Steele and McDermott [87]). Some experimental studies analysing the attraction of vectors to amphibian vocalizations showed that biting midges and Culicidae were lured to birdcalls used in control treatment [42,88]. For example, 60% of female *Culex territans*, a mosquito species that occasionally feed on birds [89], oriented themselves towards broadcasted calls from sparrows, finches, grosbeaks, and buntings in choice assays, which suggest an attraction for ectoparasites to bird songs and calls [42]. Nevertheless, Tomás et al. [43] recently investigated the attraction of different groups of blood-feeding insects to auditory cues produced by birds. They played back a recording of begging calls of hoopoe (*Upupa epops*) nestlings as auditory stimulus, but they did not find any empirical evidence showing that begging auditory cues would affect abundance of mosquitoes, blackflies, or biting midges. However, in this latter study, traps from both experimental (speakers with hoopoe begging calls) and control treatments (speakers with no sound) were also baited with a source of CO_2_. Because CO_2_ is an important cue for mosquitoes that elicits the activation and attraction towards vertebrate hosts [90], the similar abundance of vectors captured in traps from both treatments could be explained by the attraction of mosquitoes to CO_2_ baits.

### 2.4. Olfactory Stimuli and the Role of Feather/Skin Microbioma

Olfaction is a type of chemoreception in which female mosquitoes deal with volatile compounds in their environment for host detection. Air-borne chemical signals are detected by vector Odorant Receptors Neurons (ORNs) that are encased in sensory organs called sensilla. These sensilla are distributed on the tissues of olfactory appendages in the mosquito head involved in olfactory sensing: the antenna, the maxillary palp, and the labellum [90]. Cues from volatile compounds are further integrated and processed in the brain with additional information from other senses (vision, temperature, and humidity), and trigger a behavioural output leading to host finding [91].

Carbon dioxide (CO_2_) is exhaled by all vertebrates. It is considered a universal attractant and the most relevant olfactory stimulus for host-detection in mosquitoes [90]. ORNs are extremely sensitive to CO_2_, since they can detect minimum changes in CO_2_ concentrations in relation to background concentration (as low as 0.01%) [92]. It has been shown that CO_2_ can activate resting mosquitoes [61], and drive attraction and orientation at long-range (distances >1 m). In addition to mosquitoes, other biting insects are also capable of detecting CO_2_, including Simulidae and Ceratopogonidae [93]. For example, Castaño-Vázquez et al. [44] explored whether biting midges may use differences in carbon dioxide (CO_2_) concentration to locate their hosts. By analysing the temporal variation in the concentration of carbon dioxide inside nest boxes of blue tits (*C. caeruleus*) during the nestling period (from day 3 to 21 post-hatching), they showed that biting midge abundance was positively related to differences in CO_2_ between nest and forest air at day 20 of nestling age.

Once the vector has approached to host at intermediate-close range (distances <1 m), they fly around the host looking for the most attractive body part on which to land [94]. Host-volatile compounds mediate this short-range attraction [59,60]. Resident skin microbiota plays an essential role in host odour production and can affect the attraction of mosquitoes to their hosts [91,95]. In mammals, sweat is produced on the skin by eccrine, apocrine, and sebaceous glands. Secretions from these glands mainly consist of salts, proteins, amino acids, urea, ammonia, lipids, steroids, proteins, and L-lactic acid. Skin bacteria, such as *Staphylococcus*, *Propionibacterium*, and *Corynebacterium*, transform these secreted metabolites into sulphur products, aldehydes, ketones, alkenes, alcohols, carboxylic acids, and other compounds that confer the characteristic body scent and may act as attractants to vectors (see recent reviews in [34] and [91] for a detailed description of main attractants). For example, specific carboxylic acids and sulphur compounds from human skin odour are key landing cues for *Culex quinquefasciatus* [94]. In addition, nonanal, which is highly abundant in human skin odour, synergizes with CO_2_ in attracting this mosquito species to traps [96].

Mosquitoes can exhibit species-specific attraction to their hosts. These preferences seem to be triggered by host-skin volatiles and the sensitive of ONRs to such volatiles. For example, *Ae. aegypti* shows stronger attraction to specific compounds abundant in the human scent, such as ketones (sulcatone and geranylacetone) and long-chain aldehydes (decanal). This could explain the higher attraction of *Ae. aegypti* to humans compared to other animals, with greater abundance of short-chain aldehydes (hexanal and heptanal) in their scents. Remarkably, this host preference may act even at subspecies level, as the different host preferences by *Ae. aegypti* subspecies have shown. The domestic subspecies *Ae. aegypti aegypti* exhibits anthropophilic behaviour, whereas forest-dwelling subspecies *Ae. aegypti formosus* shows a strong preference to feed on wild animals. Sulcatone is a skin-emanating volatile involved in differences in host preferences between *Ae. aegypti* subspecies. This compound is present in much greater amounts in human skin volatiles than in the scent of other animals. In addition, the ORNs of the anthropophilic subspecies display a higher sensitivity to sulcatone than do the olfactory neurons of the zoophilic subspecies [97].

Symbiotic microbial bacteria have been also found in the skin and feathers of bird species [95]. *Staphilococcus*, *Bacillus*, *Lactococcus*, *Pseudomonas*, and *Stenotrophomona* are some of the most common bacteria genera found on the bird plumage [98,99]. The abundance and composition of this microbiota may differ between individuals and species. For instance, Engel et al. [100] collected skin microbe samples from three different estrildid finch species sharing the same environment and with similar diets (zebra finch *Taeniopygia guttata*, diamond firetail *Stagonopleura guttata*, and Bengalese finch *Lonchura striata domestica*) to characterise the skin microbes and compare the bacterial composition. They found significant quantitatively and qualitatively differences in the skin microbe composition among the three species.

Similar to mammals, these symbiotic microbial communities in the skin and feathers of birds play a significant role in the production of volatile compounds with vector attractant properties, such as aldehydes, alcohols, and carboxylic acids [101,102]. Hence, the odour production through the generation of these volatile compounds by symbiotic bacteria develops the characteristic scent profile for each individual [103,104], which can lead to differences in mosquito attraction to their hosts and regulate the epidemiology of vector-borne diseases [95].

## 3. The Role of Uropygial Gland Secretion in Bird–Haemosporidian Vector Interactions

The uropygial gland (also called oil or preen gland) is an epidermal holocrine gland located at the dorsal base of the tail and present in all embryonic stage bird taxa, but degenerates in some adult birds such as Amazon parrots, ostriches, and some species of pigeons and doves [105,106]. It anatomically comprises the stratified epithelium, which contains secretory tubules filled with oil droplets that are in two similar size lobules, which drain into a single small papilla [107]. The uropygial secretion is a thick, transparent, complex oil (preening oil) that is spread on feathers and skin during preening [108]. The gland is covered by a tuft of down feathers, which may help in transmitting oil from the gland to the beak while preening [109] and facilitate perception of individual odour by conspecifics [110].

The uropygial gland secretion is a complex and variable mixture of chemical compounds. Lipids are the main components of preen oil, with a proportion of 59% of unsaturated fatty acids (mainly oleic acid), where saturated long chain fatty acids are in a percentage of approximately 34% [111,112]. Compounds of the preen oil are classified according to the size of the carbon chain as volatile (short-chain) or non-volatile (long-chain) [113]. The composition of uropygial gland secretion varies between and within species [114,115,116]. In addition to lipids, other substances, such as carotenoids, could be also present in the uropygial secretion of some species such as flamingos [117].

These compounds of preen secretions show singular properties, which has been associated with the different functionalities of uropygial secretions (see reviews in [108,112,113,118]). For example, lipids may constitute a waterproofing layer improving water repellence of feathers [108,119,120]. In addition, uropygial gland secretion may hold feather microstructure, which is necessary for keeping the plumage waterproof [113,121]. Moreover, volatile components may be implied in olfactory communication [122,123,124]. Furthermore, uropygial secretion may show antibacterial and antifungal properties and thus act as defensive barrier of skin and plumage. This antimicrobial function may be conferred by microbicidal activity of some uropygial gland chemical compounds [125,126,127,128,129,130,131] or by facilitating the growth of symbiotic feather bacteria that can defeat microbial antagonists [125,127,132,133,134]. Other proposed functions for uropygial secretion include drag reduction by facilitation of air flow during flight [135], excretion of pollutants [112,136], intensification of feather coloration for colour-mediated intraspecific communication [117,137], and lessening of the effects of oil contamination [138].

### 3.1. Uropygial Gland Secretion and Vector Attraction

It has been hypothesized that the uropygial gland secretion may affect the interaction between birds and their vectors [46,48]. However, despite the increasing interest on uropygial secretion investigations in the last five years, the number of studies aiming to test the possible role of the uropygial gland in host–parasite relationships is still low and has produced mixed and inconclusive results. On one hand, it has been proposed that haemosporidian vectors are attracted by the secretions of the uropygial gland. This assumption is based on the presence in the preen oil of some volatile compounds that can be used by vectors for host searching, such as alcohols, aldehydes and waxes [34,91]. In support of this hypothesis, several studies have shown an enhanced attraction of blood-feeding dipterans to uropygial secretions. For example, Lowther and Wood [45] documented that some species of simulids were intensely attracted to uropygial gland extracts from common loon (*Gavia immer*). Likewise, Fallis and Smith [46] used uropygial secretion of common loons and carbon dioxide to experimentally test the black fly attraction to ducks. They revealed that simulids were highly attracted to a combination of extract of the uropygial glands and CO_2_, and to a lesser extent to CO_2_ alone, but not to the extract alone. Furthermore, Bennett et al. [47] effectively used ether extract of the uropygial gland of the common loon to attract simulids. Furthermore, Russell and Hunter [48] experimentally showed that the addition of cotton swabs coated with uropygial gland secretions from American crows (*Corvus brachyrhynchus*) to CDC traps captured more *Culex* mosquitoes than blank control traps without uropygial secretion.

Moreover, some other studies failed to find any evidence supporting the attraction role of uropygial secretions to blood-sucking insects. For example, Allan et al. [49] studied the attraction of several mosquito species (*Cx. quinquefasciatus*, *Culex tarsalis*, *Culex nigripalpus*, and *Ae. aegypti*) to avian and other host odours in a dual port olfactometer, but none of the tested uropygial gland compounds were attractive to mosquitoes. Furthermore, Martínez de la Puente et al. [50] experimentally tested whether biting midges and black flies were attracted to uropygial secretions from blue tits and feral pigeons (*Columba livia*). They found no difference in the number of Culicoides and simulids captured in nest boxes and CDC traps baited with preen oil and in those without secretions. Similarly, Díez-Fernández et al. [51] used a dual-choice olfactometer to analyse the behavioural response of the ornithophilic *Cx. pipens* and mammophilic *Aedes* (*Ochlerotatus*) *caspius* mosquitoes to uropygial secretions of house sparrows. None of the mosquito species showed a differential attraction towards uropygial secretions (olfactometer port baited with CO_2_ and uropygial secretion) when compared to control groups (olfactometer port baited with CO_2_ alone). Overall, these results did not support the potential role of preen oil in attracting haemosporidian vectors. Discrepancies in the outcomes found on the attraction of insect vectors to uropygial secretions can be attributed to methodological differences between studies. For example, some studies differ in the compounds used to lure insects [45,46,47,48,49], which can vary in their vector attraction [116]. In addition, studies used uropygial secretions from different bird species [45,46,47,48,50], which may vary in their composition [114,115]. Moreover, variations in the height in which insect traps were placed and differences in the light source used in CDC traps can also determine the number of captured insects [48,50]. Furthermore, the diverse insect species used in these studies [45,46,47,48,49,50] may show differences in their attractiveness towards avian odours [49].

### 3.2. Uropygial Gland Secretion May Prevent Acquiring Malaria Infection

On the other hand, some other studies have reported a negative association between the uropygial gland volume and antibacterial activity of its secretion and the haemosporidian infection, suggesting that uropygial secretions may prevent birds from acquiring blood parasite infection. In this line, Magallanes et al. [139] explored the relationship between uropygial gland size and the antimicrobial capacity of its secretions and blood parasite infection in house sparrows. Their outcomes revealed that sparrows with larger uropygial glands and/or higher antibacterial activity of their secretions have lower probabilities of being infected with haemosporidian parasites. Similar results were reported by Marzal et al. [140] when testing whether uropygial gland secretions may have promoted the establishment of invasive house sparrows in Peru. They found that uninfected sparrows had larger uropygial glands and higher anti-bacterial activity than malaria-infected house sparrows. More recently, Magallanes et al. [141] analysed the size of the uropygial gland of more than 1700 individual birds belonging to 36 bird species from neotropical and temperate areas, showing that species with larger uropygial glands for their body size have lower mean prevalence of haemosporidian infection, regardless of their geographical origin.

All these results provide evidence suggesting that preen gland secretions may reduce the likelihood of becoming infected with haemosporidians. Several mechanisms have been proposed to explain the role of uropygial gland secretions preventing haemosporidian infections. First, the antimicrobial properties of uropygial secretions may prevent acquiring haemosporidian infections by reducing the attraction of blood-feeding vectors. Ectoparasite vectors, including mammalophilic and ornithophilic mosquitoes, rely on odorant and volatile organic chemicals produced by skin and plumage bacteria to locate their hosts [57,142]. Among other functions, uropygial secretions have been proposed to have antimicrobial and antifungal properties, thus acting as a defensive barrier of skin and plumage [127,130,143,144]. For example, it has been shown that preen secretions may prevent infection by pathogenic bacteria such as *Pseudomonas* and *Staphylococcus* [145]. These bacteria are responsible of the transformation of host secreted metabolites into volatile compounds, which confer the characteristic body odour and may act as attractants to vectors [34,91]. Thus, the antibacterial activity of the uropygial secretion can reduce feather and skin microbiota and hence minimize the emission of chemical cues used by haemosporidian vectors. This should decrease the likelihood of being infected with these blood parasites.

Second, uropygial secretions may avoid mosquito bites by acting as a physical barrier reducing the mobility of vectors on bird feathers and skin [146]. Additionally, it has also been proposed that waxes from uropygial secretion may form a physical barrier preventing microbes from getting access to feather surface [126,129], which could impair the production of odour attractants.

Third, preen oil may have insecticidal properties [108]. In this sense, secretions could act as an insecticide and affect ectoparasites by covering the surface of the vector or blocking their spiracles and suffocate them [147]. In support for this hypothesis, several compounds of uropygial secretions have been identified with potential deleterious effects on vectors (see review in [32]). For example, 2-tridecanone and hexadecanoic acid have an insecticidal effect [148,149] and they have been found on the preen secretions of many bird species, including grey catbird (*Dumetella carolinensis*), Japanese waxwing (*Bombycilla japonica*), dark-eyed junco (*Junco hyemalis*), spotless starling (*Sturnus unicolor*), budgerigar (*Melopsittacus undulatus*), and white-throated sparrow (*Zoonotrichia albicollis*) [122,123,150,151,152].

Finally, it has been also hypothesized that uropygial secretions may include chemicals with arthropod repellent properties [153]. Some biochemicals with mosquito repellent activity, such as tetradecanoic acid [154] and hexadecanoic acid [149] are commonly found in uropygial secretions of some bird species, such as grey catbird, Bohemian waxwing (*Bombycilla garrulus*), Japanese waxwing, budgerigar, and dark-eyed junco [122,123,150,155]. Although some experimental tests did not find any repellence effect of uropygial secretions on mosquitoes [51,54], results from field studies support the role of preen secretions as vector repellent. In this sense, Tomás et al. [43] explored the attraction of different hematophagous ectoparasites (mosquitoes, blackflies and biting midges) to uropygial secretions and symbiotic bacteria isolated from the secretion from hoopoes, revealing that uropygial secretions and symbiotic bacteria living in this secretion may act as blood-feeding vector repellent.

## 4. Do Bird Malaria Parasites Change the Host Attractiveness to Vectors?

The *host manipulation* hypothesis (also named the *parasite manipulation* hypothesis) states that parasites can modify the behaviour, appearance, and physiology of their hosts to increase their transmission success and, thereby, their fitness [156,157]. Hence, parasites able to manipulate their vector and/or vertebrate hosts to enhance their transmission should be favoured by natural selection [158].

### 4.1. Manipulation of Vector to Increase Parasite Transmission

According to *host manipulation* hypothesis, vector-borne parasites may induce changes in phenotypic traits of their vectors to increase their transmission rates to the non-arthropod host [159,160]. In haemosporidian-vector systems, behavioural and physiological alterations in the arthropod vector induced by malaria parasites have been frequently reported. These changes include a more persistent host-seeking behaviour and feeding persistence, longer duration of mosquito bites and increased mosquito biting rate (see review in [159] and [161]). For example, it has been shown that *Plasmodium* spp. impaired the salivary function in sporozoite-infected mosquitoes by decreasing the activity of the apyrase salivary protein (enzyme with anticoagulatory properties) [162]. These malaria-induced changes can minimize the vector’s ability to engorge and hence induce infected mosquitoes to feed several times on vertebrate hosts to obtain the same amount of blood. This hypothesis was experimentally tested in birds by Rossignol et al. [163], showing an increased daily biting rate of *Ae. aegypti* mosquitoes infected with *Plasmodium gallinaceum* sporozoites (the transmission stage of the malaria parasite) compared to non-infected mosquitoes. In addition, Cornet et al. [164] monitored the effect of infection with avian malaria *P. relictum* on the blood feeding behaviour of *Culex pipiens quinquefasciatus* mosquitoes, showing that sporozoite-infected vectors completed their blood meal later and ended up with smaller blood meals than uninfected mosquitoes.

Furthermore, parasites would optimize their transmission rates favouring vector encounters with suitable hosts. Hence, a parasite manipulation of vector feeding preferences towards infected hosts should be expected (see review in [24]). In support for this idea, Yan et al. [53] found a higher feeding preference of *Cx. pipiens* mosquitoes on house sparrows naturally infected with malaria than in birds with experimentally reduced infection. In addition, Díez-Fernández et al. [54] showed that nulliparous (e.g., uninfected mosquitoes without previous access to blood) *Cx. pipiens* females were more attracted towards the whole-body odour (headspace) of *Plasmodium*-infected house sparrows than to uninfected birds in a dual-choice olfactometer. However, no enhanced attraction of vectors towards *Plasmodium* infected birds [56] or even a decreased attractiveness of infected hosts to vectors has also been found. In this line, Tomás et al. [37] experimentally reduced haemosporidian parasitaemia in female blue tits, showing a higher abundance of biting midges in nests attended by these medicated females than in control nests cared by females with higher blood parasitaemias. Similarly, it has been documented that malaria infected great tits (*P. major*) were less attractive to *Cx. pipiens* mosquitoes [55]. These results suggesting a preference of haemosporidian vectors towards uninfected birds or hosts less infected with blood parasites could be explained by the detrimental effect of haemosporidian infection on the survival of their insect vectors. For example, Valkiūnas and Iezhova [165] reported higher mortality rates in biting midges *Culicoides impunctatus* experimentally infected with *Haemoproteus* than in uninfected control vectors. Likewise, Gutierrez-López et al. [166] experimentally reduced *Plasmodium* parasitaemia in house sparrows with an anti-malaria treatment, showing that the mosquitoes that fed on medicated birds had a higher lifespan than those that fed on control sparrows.

### 4.2. Manipulation of Vertebrate Host Attractiveness to Vectors

The feeding preference of haemosporidian vectors to infected hosts and/or hosts infected with transmissible stages of malaria leads to a more successful parasite transmission, which is in accordance with the parasite manipulation hypothesis. Although host attractiveness could be modified by the parasite, the definitive effect is the alteration of mosquito behaviour, which subsequently increases parasite transmission to the vector. Because host-seeking behaviour is mainly driven by a set of different stimuli [142], the question arisen from here is whether parasites may alter the host attractiveness to vectors by changing the appeal of cues followed by blood-sucking insects to detect their hosts.

Some studies have proposed that some clinical symptoms of malaria infection, such as fever and the increased production of sweat due to fever episodes, could guide *Anopheles* mosquitoes in host-seeking towards *Plasmodium*-infected humans [167,168]. Host-seeking behaviour in haemosporidian vectors is mainly prompted by olfactory perception of volatile organic compounds (VOCs) emitted by hosts [91]. Changes in VOCs profile during infection likely constitute the most important factor determining vector attraction. Therefore, *Plasmodium* parasites could increase the infected host attraction to mosquitoes by manipulating host-VOC profiles [169]. In agreement with this hypothesis, it has been documented that children suffering from high malaria parasitaemia produce larger amount of mosquito attractant VOCs (heptanal, octanal, nonanal, (E)-2-octenal, (E)-2-decenal, and 2-octanone) on their skins than patients having either low malaria parasite density or being parasite-free [170]. In addition, Schaber et al. [171] showed that children with malaria have a distinct shift in overall breath composition (higher breath levels of 2 mosquito-attractant terpenes, α-pinene, and 3-carene). In birds, Grieves et al. [172] compared the chemical profiles of uropygial secretion from song sparrows (*Melospiza melodia*) before and 13 days after malaria inoculation (corresponding to peak infection intensity), showing that wax ester profiles of uropygial secretion varied in sparrows that became acutely infected, but not in sham-inoculated control individuals. Contrasting results were found by Díez-Fernández et al. [173] when evaluating whether the chemical composition of uropygial secretions is associated with malaria infection in house sparrows. By using gas chromatography-mass spectrometry analyses, they found no significant differences in the composition of the volatile lipophilic components in the uropygial secretions of infected and uninfected house sparrows.

Skin and feather bacteria are responsible for the transformation of sweat components to VOCs [174]. Because the presence of blood parasites may modify the odour of an individual by altering the profile of symbiotic microbial community [175], the infection with malaria parasites may result in increased attractiveness of hosts. In this sense, an increased attractiveness of malaria-infected hosts to mosquitoes has been shown in humans [170,174,176,177], rodents [178,179], and birds ([52,54]; see review in [180]). However, to date there are no empirical studies linking malaria infection with changes in feather, skin, or preen gland microbiota and vector attraction. In birds, Videvall et al. [181] recently found that house sparrows infected with malaria harboured significantly higher abundances of bacteria from the genera *Arthrobacter* and *Micrococcus* in their uropygial gland, whereas uninfected sparrows had higher abundances of *Rhodococcus*, *Phenylobacterium*, and *Enhydrobacter*. These outcomes suggest a specific association between some symbiotic bacteria of the uropygial gland microbiota and *Plasmodium* parasites in birds, highlighting new questions on the role of the uropygial gland in host–parasite interaction.

## 5. Conclusions and Future Lines of Research

Vector-borne infectious diseases, such as malaria, represent one of the most critical concerns facing public health systems. Since the discovery of the mosquito transmission of malaria in birds in 1897, investigations on avian malaria have allowed significant advances to understand the dynamics and mechanisms of vector-borne disease transmission, but many challenges remain to be overcome.

Vector host-seeking behaviour is a key determinant of pathogen transmission and the epidemiology of vector-borne diseases. Therefore, studies aiming to understand stimuli used by vectors to locate their hosts will provide valuable information for vector surveillance and control policies. Here we have reviewed the role of physical and chemical stimuli used by haemosporidian vectors to detect their avian hosts. Volatile compounds emitted by avian hosts (e.g., CO_2_) are the main olfactory stimuli for attraction and orientation of vectors at long-range distances. In addition, host-odours produced by skin and feather microbiota include volatile compounds with vector attractant properties that may mediate host preferences by vectors. Moreover, host body size, contrasting colours against dark background, and motion activity have been also suggested to act as visual cues for avian haemosporidian vectors to locate their hosts. Other physical cues, such as heat and moisture, can also be perceived by insects and be used to identify the presence of hosts in short-range, but the outcomes from different studies have revealed mixed results on the role of host temperature and humidity in vector attraction. Likewise, studies analysing the capacity of insect vectors to use bird sounds and calls for host location are still rare and have thrown inconclusive results. Similarly, the role of the uropygial gland in the interaction between birds and their vectors remains quite unexplored. Some studies have proposed that haemosporidian vectors are attracted to preen gland secretions, whereas results from recent studies suggest that uropygial gland secretions may reduce the attraction of blood-feeding vectors and prevent acquiring haemosporidian infection. In sum, these outcomes suggest that physical and chemical cues can be used simultaneously by vectors, and integration of different stimuli is required for accurate host location. The discrepancies in the outcomes found on the attraction of insect vectors to different stimuli can be attributed to methodological differences between studies, or to variations among vector species in the attraction to different hosts.

Pathogens may induce changes in the host seeking and feeding behaviours of their vectors, or manipulate host attractiveness to vectors, hence enhancing parasite transmission. The comprehension of these behavioural alterations would promote a complete understanding of vector-borne disease systems and a full depiction of transmission dynamics. Moreover, the knowledge of the types of behavioural shifts induced by haemosporidian parasites could help to identify suitable targets for malaria control. For example, symbiotic bacteria species and the microbiome composition of skin, feathers, and preen gland could be controlled to minimize vector attraction and hence pathogen transmission to hosts. Moreover, further studies exploring the potential role of uropygial secretions and symbiotic bacteria living in this secretion as blood feeding vector repellent or insecticide may provide important advances for vector-borne disease mitigation efforts. Furthermore, studies on human and other animal models have revealed that malaria infection may alter host odours that influence vector attraction, suggesting that these volatile biomarkers may have significant potential for the development of next generation screening methodologies for malaria identification and infectious disease management.

## Figures and Tables

**Table 1:** Summary of studies reporting increased (+), decreased (−), or neutral (0) attraction of avian haemosporidian vectors towards different stimuli.

Stimulus		Host	Vector	Effect	Explanation	Reference
Visual	Colour	49 North American bird species	*Culex pipiens*	+	Mosquitoes fed preferably on birds with lighter-coloured plumage.	[36]
	Motion	*Cyanistes caeruleus*	Biting midges	+	Abundance of biting midges was positively associated with parental provisioning effort (increased motion activity).	[37]
	Size	49 North American bird species	*Culex pipiens*	+	Mosquitoes fed preferably on birds with longer tarsi.	[36]
Heat and moisture	Temperature	*Ficedula hypoleuca*	Biting midges	+	Abundance of biting midges increased with temperature inside the bird nests.	[38]
	Temperature	*Parus major*	*Culex pipiens*	−	Birds with a lower body temperature were preferentially chosen by mosquitoes.	[39]
	Metabolic rate	*Passer domesticus*	*Culex pipiens*	−	House sparrows with lower metabolic rate suffered more mosquito bites.	[40]
	Moisture and temperature	*Cyanistes caerules*	Biting midges and black flies	0	No higher abundance of biting midges and black flies in nests with higher temperature and lower humidity.	[41]
Acoustic	Bird calls	*Passer, Fringila, Emberiza*	*Culex territans*	+	60% of female mosquitoes oriented toward the bird songs in phonotaxis experiments.	[42]
	Auditory stimulus	*Upupa epops*	Mosquitoes, blackflies and biting midges	0	Auditory cues of nestling hoopoes did not affect the abundance of vectors.	[43]
Olfactory	Carbon dioxide (CO_2_)	*Cyanistes caeruleus*	Biting midges	+	Higher biting midge abundance in nests boxes with CO_2_ levels higher than in the forest air.	[44]
Uropygial gland secretions	Uropygial secretion	*Gavia immer*	*Simulium euryadminiculum*	+	Black flies were attracted to the odour of the common loon’s uropygial gland.	[45]
	Uropygial secretion	*Gavia immer*	*Simulium euryadminiculum*	+	Higher attraction of black flies to a combination of ether extract of the uropygial glands and CO_2_ than to CO_2_ alone.	[46]
	Ether extract	*Gavia immer*	*Simulium euryadminiculum*	+	Black flies were attracted to ether components of the uropygial gland.	[47]
	Cotton swabs coated with uropygial secretions	*Corvus brachyrhynchus*	*Culex pipiens, Culex restuans*	+	CDC traps baited with uropygial secretions captured more mosquitos than control traps.	[48]
	Diol volatile compounds from Natasauropygial gland secretion		*Culex quinquefasciatus Culex tarsalis, Culex nigripalpus, Aedes aegypti*	0	Meso-2,3-butanediol, 2,3-butanediol, and 2,3- docosanediol were not attractive to mosquitoes.	[49]
	Uropygial secretions	*Columba livia Cyanistes caeruleus*	Biting midges and black flies	0	No differences in the number of vectors captured in CDC traps or nests with this stimulus.	[50]
	Uropygial secretions	*Passer domesticus*	*Culex pipiens, Aedes caspius*	0	Mosquitoes were attracted equally to the ports containing uropygial secretion and to the control in olfactometer assays.	[51]
	Uropygial secretions	*Upupa epops*	Biting midges	−	Traps baited with uropygial secretion in pine forest significantly captured less biting midges than control traps.	[43]
Haemosporidian infection	Bird infected with malaria	*Serinus canaria*	*Culex pipiens*	+	Chronically infected birds attracted more vectors than either uninfected or acutely infected birds.	[52]
	Bird infected with malaria	*Passer domesticus*	*Culex pipiens*	+	Higher feeding preference of mosquitoes on infected sparrows.	[53]
	Bird infected with malaria	*Passer domesticus*	*Culex pipiens*	+	Mosquitoes were more attracted to the odour of malaria-infected sparrows.	[54]
	Bird infected with malaria	*Cyanistes caeruleus*	*Biting midges*	−	Higher abundance of biting midges in the nest attended by medicated birds with reduced parasitaemia.	[37]
	Bird infected with malaria	*Parus major*	*Culex pipiens*	−	Plasmodium-infected birds attracted significantly fewer mosquitoes than the uninfected ones.	[55]
	Bird infected with malaria	*Corvus monedula Passer domesticus*	*Culex pipiens, Aedes caspius*	0	Similar biting rates of mosquitoes on malaria infected and uninfected birds.	[56]

## Data Availability

Data sharing is not applicable to this article.
